# Advances in Near-Infrared Spectroscopy and Related Computational Methods

**DOI:** 10.3390/molecules24234370

**Published:** 2019-11-29

**Authors:** Krzysztof B. Beć, Christian W. Huck

**Affiliations:** Institute of Analytical Chemistry and Radiochemistry, CCB-Center for Chemistry and Biomedicine, Leopold-Franzens University, Innrain 80/82, 6020 Innsbruck, Austria

Over the last few decades, near-infrared (NIR) spectroscopy has distinguished itself as one of the most rapidly advancing spectroscopic techniques [1]. Mainly known as an analytical tool useful for sample characterization and content quantification, NIR spectroscopy is essential in various other fields, e.g., NIR imaging techniques in biophotonics, medical applications, or in characterization of food products, to name the few [2]. Its contribution to basic science and physical chemistry should be noted as well, e.g., in exploration of the nature of molecular vibrations or intermolecular interactions [3]. One of the current development trends involves the miniaturization and simplification of instrumentation [4], creating prospects for the spread of NIR spectrometers at a consumer level, e.g., in the form of smartphone attachments—a breakthrough not yet accomplished by any other analytical technique. NIR spectroscopy has been developing in conjunction with advanced methods of data analysis; recent years have highlighted the role of anharmonic quantum mechanical computations in shedding light on the complex nature of NIR spectra as well [5].

The importance of NIR spectroscopy is well demonstrated by a remarkable interest it receives among scientific and professional communities. Such observation can be roughly quantified using the statistical data collected by Web of Science [6]. A query for “near infrared spectroscopy” returns over 2200 records for 2018 year alone, out of this number almost 1800 records being scientific articles (Figure 1). This clearly evidences the maturity level that NIR spectroscopy has achieved nowadays. At the same time, statistical data evidences a steady progress in popularity of the eponymous technique as the number of articles published annually almost doubled over the last decade comparing 2009 to 2018 (Figure 1). However, one may also notice some adverse effects of such popularity. As unveiled by Web of Science query, this technique is used throughout various fields of application in a true myriad of contexts (Figure 2) [6]. The mentioned trend also resulted in a growing diversity of the methods and applications related to NIR spectroscopy and has led to a dispersion of the contributions among disparate scientific communities.

For this reason, we recognized the need to propose the Special Issue “Advances in Near Infrared Spectroscopy and Related Computational Methods” in *Molecules* journal. Our aim was to bring together these diverse communities, which may perceive NIR spectroscopy from different perspectives. Besides, we welcomed research topics not directly focused on the NIR region, however, which remained relevant by employing the methodologies essential in NIR spectroscopy. A number of other spectroscopic methods of analysis share methods and tools common with NIR spectroscopy. We believe such scope of the Special Issue promoted the exchange of ideas and thus was helpful in pushing the frontier of this discipline of science. Moreover, we hoped to create a formidable opportunity for the readership to obtain a thorough overview of state-of-the-art NIR spectroscopy, current development trends, and future prospects with no artificial limits or strict categorization. This way, we also put faith in offering an appropriate opportunity to all the contributors to make their results and techniques more visible, and to present the most recent accomplishments in their respective fields that have become possible with the use of NIR spectroscopy. 

The Special Issue has met a remarkably positive feedback with many contributions submitted by numerous scholars and professional spectroscopists performing their active research in academia and industry, resulting in a collection of 30 publications including two exhaustive review articles [7,8,9,10,11,12,13,14,15,16,17,18,19,20,21,22,23,24,25,26,27,28,29,30,31,32,33,34,35,36]. The diversity in the application field has been well represented by the submitted manuscripts. These articles discuss a variety of aspects relevant to NIR spectroscopy in a markedly broad context. 

Many of these articles have a cross-field character and it would be difficult to ascribe them arbitrarily to certain disciplines of science. However, for sake of clarity a tentative and brief overview of these contributions may be helpful to present the Special Issue to the readership. The majority of the articles focuses on applied qualitative and quantitative analyses in a variety of fields [9,11,12,17,18,19,20,21,22,23,24,25,26,27,28,29,30,31,32,33,34]. Roughly, half of these may be associated with pharmaceutical and medical applications [17,18,19,20,21,22,23,24]. Most of the remaining applied studies were directed at agricultural applications [25,26,27,28,29,30,31,32,33,34], well reflecting the ever-growing significance of NIR spectroscopy in this area; a good perspective of this topic is included in a focused review article published in the Special Issue [30]. Modern strategies for food analysis also rely on this technique, and few contributions touched that field as well [9,12,33,34]. State-of-the-art analytical spectroscopy is based on sophisticated data-analytical methods. Development of new methods is, therefore, essential and benefits multiple applications [10,11,13,14,15,16]. Several articles focused on this direction, and the importance of research and development of calibration transfer methods is well reflected in this Special Issue [13,16]. Interestingly, Beganović *et al.* demonstrated that there exists room for improvement in fundamental aspects of analytical spectroscopy such as wavenumber region selection for subsequent calibration [3]. On the other hand, progress in technology and instrumentation is indispensable as well. The growing applicability and importance of miniaturized, portable NIR spectrometers is reflected by several focused articles [10,11,12]. The differences in design principles and emerging novel technologies that become applied in order to obtain affordable and ultra-miniaturized devices raise concerns about the resulting analytical performance of such spectrometers; therefore, comparative evaluation studies are critical [11,12]. Likewise, the potential of hyperspectral imaging can be recognized on the basis of the articles collected in this Special Issue as well [29,30,31,32]. The importance of NIR spectroscopy as a potent tool in exploring the complex nature of water, the elementary substance, is reflected in an exhaustive review article [36]. Finally, contributions focused on fundamental principles of NIR spectroscopy including theoretical NIR spectra simulation and physicochemical research should be mentioned, highlighting the significance of pushing the frontier of the underlying basic science [7,8]. One may note that these contributions reflect well the diversity and dynamics of contemporary development trends in NIR spectroscopy.

This special issue is accessible through the following link: https://www.mdpi.com/journal/molecules/special_issues/infrared_computational

As Guest Editors for this Special Issue, we would like to thank all the authors and co-authors for their contributions and all the reviewers for their effort in carefully evaluating the manuscripts. Last but not least, we would like to appreciate the editorial office of *Molecules* journal for their kind assistance in preparing this Special Issue.

## Figures and Tables

**Figure 1:**
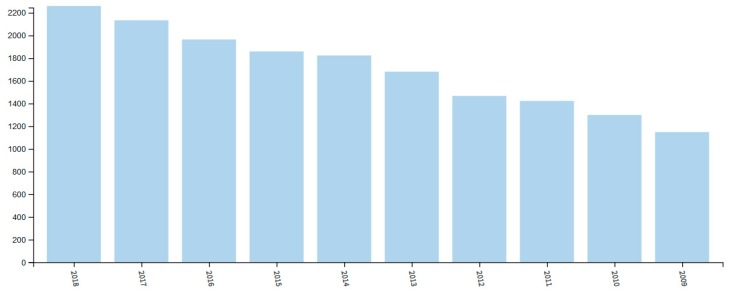
Results analysis for Web of Science query “near infrared spectroscopy” for publication years (2009–2018) [6].

**Figure 2:**
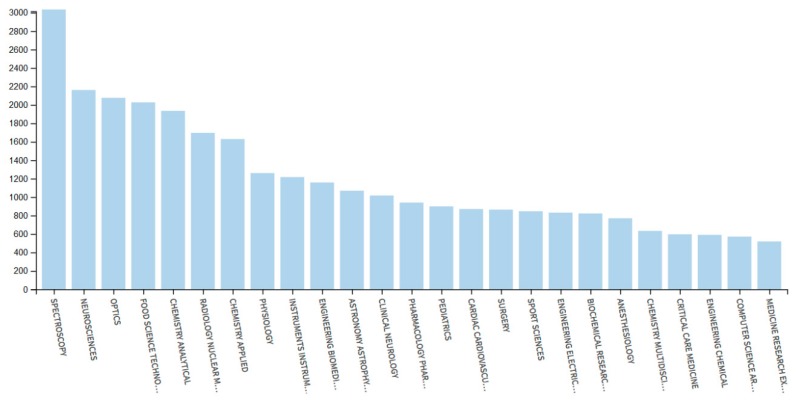
Results analysis for Web of Science query “near infrared spectroscopy” following the classification of Web of Science Categories. The figure presents only the 25 most significant categories [6].
